# Use of traditional medicine for the treatment of diabetes in Eastern Uganda: a qualitative exploration of reasons for choice

**DOI:** 10.1186/1472-698X-13-1

**Published:** 2013-01-02

**Authors:** Elizeus Rutebemberwa, Muhamadi Lubega, Sheila K Katureebe, Abanga Oundo, Francis Kiweewa, David Mukanga

**Affiliations:** 1 Department of Health Policy, Planning and Management, Makerere University School of Public Health, Kampala, Uganda; 2 Iganga-Mayuge Demographic Surveillance Site, Iganga, Uganda; 3 African Field Epidemiology Network, Kampala, Uganda; 4 Iganga Hospital, Iganga, Uganda; 5 Department of Community Health and Behavioural Sciences, Makerere University School of Public Health, Kampala, Uganda; 6 Bugiri Hospital, Bugiri, Uganda; 7 Joint Clinical Research Centre, Kampala, Uganda; 8 Division of Global Health, IHCAR, Department of Public Health Sciences, Karolinska Institutet, Stockholm, Sweden

## Abstract

**Background:**

While there are biomedical drugs for managing diabetes mellitus, some patients with diabetes use traditional medicine. The aim of the study was to explore why patients with diabetes use traditional medicine for the treatment of diabetes.

**Methods:**

The study was conducted in Iganga and Bugiri districts in Eastern Uganda using four focus group discussions (FGDs) with patients with diabetes; two with female patients and two with male patients, thirteen key informant interviews (KIIs); nine with health workers working with patients with diabetes and four with herbalists. FGDs and KIIs focused on what respondents perceived as reasons for patients with diabetes taking traditional medicine. Analysis was done using content analysis.

**Results:**

Reasons for taking traditional medicine included finding difficulties accessing hospitals, diabetic drugs being out of stock, traditional medicine being acceptable and available within community, as well as being supplied in big quantities. Others were traditional medicine being cheaper than biomedical treatment and payment for it being done in installments. Traditional medicine was also more convenient to take and was marketed aggressively by the herbalists. Influence of family and friends as well as traditional healers contributed to use of traditional medicine.

**Conclusions:**

Possibilities of putting diabetic drugs at facilities closer to patients need to be considered and health facilities should have a constant supply of diabetic drugs. Community members need to be sensitized on the proper treatment for diabetes mellitus and on the dangers of taking traditional medicine.

## Background

While there are biomedical drugs for managing different types of diabetes mellitus, patients with diabetes use other complimentary/traditional herbs [1,2]. Herbs have been reported as one of the remedies used for treatment by patients with diabetes in Zimbabwe [3], Nigeria [4], Vietnam [5], Oman [6], India [7], and China [8]. The efficacy of traditional herbs in the treatment of diabetes is still mixed. Some studies have demonstrated that herbs can delay the progress of diabetic complications [8-10] while other studies have shown that certain herbs used in the management of diabetes are not efficacious [11]. Using herbs that have no proven clinical benefit to patients may lead to delays in seeking appropriate treatment leading to severe diabetes related complications and associated disability and mortality. Studies done in Canada and Brazil to explore patients preferences showed that patients preferred treatment that was less costly, had less side effects, was more convenient, and more effective [12]. There is paucity of literature on why patients with diabetes use herbs for managing diabetes mellitus in African countries. The rationale for patients’ use of these traditional herbs sometimes in the absence of proven efficacy needs to be explored. The aim of this study was to explore why patients with diabetes use traditional medicine for the treatment of diabetes.

## Methods

### Study area

The study was conducted between July and August 2011 in two government owned general hospitals – Iganga and Bugiri. They are both 100 bed general hospitals built in the 1960’s, both have diabetic clinics, and are along the Jinja – Tororo highway. Iganga hospital is 115 km eastwards from Kampala and Bugiri hospital is 32 km east of Iganga. The majority of the clientele in both hospitals were Basoga but there were also many other tribes like the Samia, the Jopadhola and the Baganda. The main economic activity in these districts is subsistence agriculture. The government owned health facilities are not supposed to charge any money from the patients [13].

Iganga general hospital acts as a referral hospital for lower level health facilities in Iganga district, the districts of Kaliro to the north, Namutumba to the north east, and Mayuge to the south. Diabetic management services at Iganga hospital were officially started in 2004. The clinic runs once a week and is managed by an assistant physician, two nurses and one nursing assistant. The services offered include random blood tests for suspected diabetic clients, health education as well as refills of diabetic drugs such as insulin and glibenclamide. The total number of clients at the clinic by the time of the study was 340, of whom 182 were females and 158 were males [14].

Bugiri general hospital acts as a referral for lower level facilities in Bugiri district, Namayunge district to the south, Busia district to the east, some parts of Tororo to the north east and the neighbouring areas in Iganga. By June 2011, it had 450 patients with diabetes in its register (282 males and 162 females). It conducted the diabetic clinic on Thursdays. Diabetic management services started in 1998 with 60 registered clients. Among the services offered were; health education on nutrition, exercise, personal hygiene, sharing of information with family members such that they are able to be of support to patients, basic investigations like testing for urine or random blood sugar and treatment of opportunistic infections. The drugs that were distributed by the clinic included insulin, glibenclamide and glucophage. There were frequent drug stock outs, lack of testing reagents, poor client adherence and heavy workload on clinic days [15].

### Study design, study population and data collection

This study explored why patients with diabetes use traditional medicine for treatment. Exploratory studies are used to understand little-known phenomena [16]. The data collection methods included four focus group discussions (FGDs) and 13 key informant interviews (KIIs). FGDs are used to explore people’s views about the issue being discussed in a certain context [17] and in this context respondents were asked why patients with diabetes use traditional medicine. An advantage of using FGDs is that the study participants are in an atmosphere more natural than artificial experimental circumstances and are more relaxed than the exposure of a one-to-one [16]. We chose to use FGDs because use of traditional medicine was a sensitive subject in this community. One-on-one interviews with patients had the possibility of introducing reporting bias. KIIs are used with well informed people to provide an overall view of the community [18] and in this context, the herbalists and health workers who interact with patients with diabetes were taken to be informed about patients’ use of traditional herbs. Two social scientists experienced in qualitative research methods and who had undergone a two days training on the study objectives and tools conducted the FGDs and KIIs. They both spoke and understood the local language Lusoga and English fluently. ER, LM and OM introduced the research assistants to the patients with diabetes but they did not sit in the FGDs so as not to make participants give biased ‘medically correct’ responses. However, ER, LM and OM kept reviewing the data being collected daily together with the research assistants. The FGDs lasted between 40 minutes to one hour. The interviews lasted between 30 to 45 minutes.

Four FGDs were conducted with patients with diabetes; two with female patients and two with male patients. Participants of the FGDs were selected sequentially as they reported for treatment. The demographic characteristics of the respondents in the FGDs are shown in Table 1. Each hospital had one FGD with male patients and one with female patients. In these hospitals, patients have appointments for the specific day but not the specific time. Patients trickle in one by one and it takes a lot of time to get eight female or male patients together. In order to gain more information, both hospitals were used to provide an FGD each. By separating women from men, women had an opportunity to openly discuss their issues something they may not have done in the presence of men [17]. Patients with diabetes were mobilized by the nurse working in the diabetic clinic. They were chosen from those who would have come to the diabetic clinic for routine check up without diabetic complications. Patients with diabetes were considered ‘information rich cases’ [19] because they would have been exposed to opportunities of using traditional medicine and there is a possibility that some of them could have used it. The FGDs were conducted in Lusoga in rooms close to where diabetic clinics were held. One research assistant acted as a note taker and the other as the moderator. The number of participants in each FGD was eight as recommended [20]. Some of the FGD questions were: what challenges do you get accessing treatment for diabetes; what is your opinion about herbs as treatment for diabetes; why do you think some patients use traditional medicine to treat diabetes.

**Table 1:** Demographic characteristics of the respondents from FGDs

**Characteristic**	**FGD 1 Women**	**FGD 2 Women**	**FGD 3 Men**	**FGD 4 Men**
**Number of participants**	8	8	8	8
**Age (years)**				
Range	32-67	15-68	30-75	16-65
Average	53.9	43.5	49.8	45.4
**Education (level completed)**				
-No formal education	3	2	0	1
-Primary level	4	5	0	5
-Secondary level	0	0	5	2
-Tertiary level	1	1	3	0

Nine KIIs were conducted with health workers working in diabetic clinics in Iganga and Bugiri hospitals. There were five nurses, three clinical officers and one medical officer. Health workers interacted with patients frequently and were expected to have perceptions on why patients of diabetes used traditional medicine. Interview questions included: which treatments do patients of diabetes get; where do they get this treatment from; and why do they use traditional medicine? Interviews were conducted in the offices of health workers in English.

Four KIIs were conducted with herbalists in their homes. Herbalists were considered as ‘information rich cases’ [19] as they prescribe herbs to patients with diabetes. They were identified and contacted by a field liaison person from Iganga district administration. They were asked to give their opinions on why patients with diabetes were using herbs for treatment.

### Data management and analysis

Both FGDs and KIIs were tape recorded and transcribed apart from one KII with a herbalist who refused to be recorded. Recording interviews provided an accuracy that would not be obtained from field notes although field notes were very useful in recording the non-verbal communication which was useful in interpreting the data. FGDs and KIIs with herbalists were conducted in Lusoga then transcribed in English by the interviewers and afterwards the tapes were cross checked by ER for completeness. ER understands both English and Lusoga. Content analysis was used. Individual FGDs and KIIs constituted the units of analysis. Passages were analysed for meaning units, then coded and emerging themes identified. An example of how the analysis was made is given in Table 2.

**Table 2:** An example of the process of analysis

**Meaning unit**	**Codes**	**Themes**
*I absconded from hospital medicine and went for herbs. The worst situation is that the herbalist stops you from taking hospital medicine and gives you hope that herbs cure diabetes.* (FGD men)	Herbalists promise cure to the patients	Belief that traditional medicine can cure diabetes
*I was told by the herbalists that their medicine could cure diabetes. I started on their treatment and I kept on taking jerry cans and jerry cans and even absconded from clinic days….* (FGD women)	Herbalists promise cure for diabetes	
*You see these people are desperate for their dear lives, so when they hear of a herbalist who heals diabetes completely, they obviously go.* (KI health worker)	Patients hear of a herbalist who can cure diabetes and they go there for treatment	

### Ethical considerations

The study was approved by Makerere University School of Public Health Higher Degrees and Research Committee and the Uganda National Council of Science and Technology. Permission to carry out the study was received from local leaders and the hospital management. Informed consent was obtained from all participants verbally and individually. At the beginning of each interview or FGD, the research assistants and participants introduced themselves and the research assistants then explained the objectives of the study. This was done in the local language for the FGDs and herbalists. Participants were told the expected benefits of the study especially for the community and assured of confidentiality and anonymity. It was made clear to all the respondents that they were free not to contribute when they felt they shouldn't and were free to withdraw any time. Respondents were assured that no services would be withheld because of their withdrawal of participation.

## Results

From the study, there were three emerging themes on why patients with diabetes used traditional medicine. (1) Easy accessibility, acceptability, availability and affordability of traditional medicine. (2) Influence from family and friends. (3) Influence from traditional healers.

### Traditional medicine was accessible, available, acceptable, met people’s expectations and was affordable

#### Patients’ focus groups

Patients with diabetes intimated that they used herbs whenever they came for treatment at the hospital and failed to get drugs.

"“Whenever I come here to get treatment and fail to get it, I use herbs; I go back home and get “Ekikaka” (aloevera), I boil it and keep it for two days, mix it with bee honey, then I take until I get biomedical drugs because you can’t just sit without taking anything.” (FGD diabetic men)"

Poor geographical accessibility was also partly responsible for patients with diabetes using herbs. Some people resort to herbs because they are unable to pay transport to go to hospital.

"“I have a friend, when I told him to leave herbs he refused. …he told me he did not have money to bring him to hospital and he said it was the reason why he was using herbs.” (FGD diabetic men)"

Traditional medicine was easy to use and could be taken concurrently with biomedical medicine.

"“The good thing with this herbal medicine is that you can take it even when you are on the biomedical dose.” (FGD diabetic women)"

#### KIIs with herbalists

Herbalists explained that they were very easy to reach and they made an effort to go to places where people can easily access them like taxi parks. This made the herbs accessible and available.

"“I am an easy person to catch and I sometimes take my medicines in the park and if you went asking for me they will tell you where I am or to tell you to wait for me in case I haven’t come at that time.” (KI herbalist)"

Payment for the herbs could be done in installments. This contrasted with biomedical doctors who at times wanted all the payment upfront.

"“If a patient has come to me for treatment, I give the medicine and the patient pays in installments because as you know this disease is very expensive as far as treatment is concerned. Most of the biomedical doctors can’t treat them without asking for money so the patient says, ‘Let me go and see the herbalist as I look for money to go and get treated by the doctor’. For me, when they come, I treat them and they pay me when they can. Actually long ago, patients paid us after getting better but now things have changed as you also know. But no doctor will treat a patient who has not paid him any money.” (KI herbalist)"

#### KIIs with health workers

The health workers concurred with the herbalists that the herbs were easy to get. They were even easier to administer as patients did not fear getting overdose like they do for biomedical drugs. While taking herbs, the patients did not need to follow strict dietary instructions. In addition, herb usage was supported by, and acceptable to community members.

"“The herbs are easy to get. …the other issue is that, there are no regulations like ours; they (patients) take any quantity they want. So, they think it is flexible; you can take herbs any time but with ours, you say first eat food before you inject yourself.” (KI health worker)"

Using both biomedical and traditional medicine was even tolerated by health workers. Health workers told the patients with diabetes that they could take the herbs as long as they were taking the biomedical medicine as well. This was an implicit permission of the health workers to concurrently use traditional herbs and biomedical medicine.

"“Some few patients have told us (that) they have been using herbs but those few who come and visit the clinic, we always tell them that; ‘we don’t refuse you to take your herbs but you can use this biomedical drugs also; combine the two’. This was because we were getting some patients who were saying that they have abandoned the tablets and injections and they were using herbs only.” (KI health worker)"

Health workers also attributed the use of traditional herbs to inability of patients with diabetes to purchase biomedical drugs. The traditional herbs were sold in jerry cans that would last a long time while biomedical medicine was in vials of insulin that would last a few days. When patients run out of money to procure the biomedical medicine, they substitute biomedical medicine with traditional herbs.

"“These patients have been purchasing these biomedical drugs from the private clinics but those who fail to get money to buy biomedical drugs, they resort to herbal treatment. Much as they also sell it (traditional medicine) expensively, at least for it they give it in big quantities. The patients take long on say the jerry can they have bought but the bottle of insulin which is at 15,000/= (about USD 6) will last just for a few days. And psychologically, for them they think it (traditional medicine) is cheaper and more affordable to them and it’s in bigger quantity.” (KI health worker)"

### Influence from family and friends

#### Patients’ focus groups

Patients with diabetes indicated that people who get treatment from herbalists recruit others in the use of herbs. One patient had this to say:

"“With herbs, I was told by the people who went to the herbalist …, I started on their treatment and I kept on taking jerry cans and jerry cans ….” (FGD diabetic women)"

#### KIIs with herbalists

Herbalists also said that the patients were referred to them by community members especially those whom the herbalist would have treated before.

"“For a patient to end up at my place for treatment will depend on who I treated .. The person I treated is the one who will refer this patient to me depending on what he saw in my herbs.” (KI herbalist)"

### Influence from traditional healers

#### Patients’ focus groups

Many patients with diabetes who reported having used traditional medicine were sometimes told that herbs would cure them from diabetes. One diabetic patient had this to say:

"“I am now admitted in the hospital because I absconded from hospital medicine and went for herbs. The worst situation is that the herbalist stops you from taking hospital medicine and gives you hope that herbs cure diabetes; herbs do not treat diabetes.” (FGD diabetic men)"

#### KIIs with herbalists

The belief in herbal cure of diabetes was reiterated by the herbalists. One herbalist reported that if the illness is still in the early stages and as long as patients take herbs according to instructions, diabetes would get cured completely.

"“What I can say is that …, I can treat diabetes. … If the person comes and the disease is still in its early stages, I treat it. …If you follow my words to the dot, you heal. You check this person and he completely has no diabetes. Yes, it will completely not be there.” (KI herbalist)"

#### KIIs with health workers

Some health workers also attributed patients’ use of traditional medicine to traditional healers who promoted their herbs as curing diabetes.

"“The reason patients with diabetes give is that this biomedical treatment doesn’t cure and the traditional healers always promise to cure them. So whenever they meet the herbalists, herbalists deceive them a lot that they abandon biomedical treatment and resort to herbal medicine. When you ask patients why they are taking herbs from the traditional healer, they tell you that the traditional healer told them that he is going to cure their diabetes.” (KI health worker)"

Use of traditional medicine by patients with diabetes demonstrates challenges patients go through and the choices patients have to make to access diabetic treatment. Patients have to travel to hospitals for treatment that is expensive, requires special dietary requirements and only controls diabetes but is not curative. Sometimes hospitals have stock outs of diabetic drugs. On the other hand, community members and some patients with diabetes find herbal treatment acceptable, accessible, affordable and easy to administer.

## Discussion

The use of traditional medicine was due to a combination of inadequacies in the functionality of the health care services, easy accessibility of traditional medicine, influence of community members and influence from traditional healers.

In this study, respondents indicated that the biomedical health care system presented challenges with long distances to health facilities which may be costly and stock outs of diabetic drugs. Other studies have highlighted challenges of inadequate infrastructure in the management of diabetes in sub-Saharan Africa. Access to facilities that can adequately manage diabetes is low [21]. Availability of drugs is also a challenge in hospitals especially for chronic diseases [22]. Health care systems in Africa are still adapting to the transition from having a high disease burden of communicable diseases to one that also has a high proportion of non-communicable diseases [21]. The issue of high access costs is complicated by the fact that compared to biomedical medicine, traditional medicine is cheaper. The herbalists even allow payment in installments which makes it easier for the patients to afford traditional medicine. Other studies have highlighted that patients with diabetes use traditional medicine due to the low cost [23]. It is costly to treat diabetes in sub-Saharan Africa [24]. Diabetes is a chronic illness and its effects on the finances of the individual and the household cannot be underestimated. If patients with diabetes are to be exhorted to use biomedical medicine instead of traditional medicine, there should be biomedical medicine at the facilities when patients come for it. This may entail more funding for the diabetic treatment. The number or patients with diabetes is increasing [25-27]. The facilities may not be adequate for the increasing number of patients with diabetes but even in those facilities which manage diabetes the supply of drugs is not regular. This calls for an increase in the number of facilities that can adequately manage diabetes possibly by putting these drugs at lower level facilities. There is also need to streamline the procurement and supply chains for diabetic drugs at all levels that manage diabetes patients to ensure a constant supply.

Findings from this study indicate that traditional herbs were easily accessible. Herbalists live within the community and sometimes bring their medicine to parks where they can be easily accessed. A systematic review on studies on diabetes in sub-Saharan Africa indicated challenges of accessing diagnosis and treatment [24]. A study done in Uganda demonstrated preferential use of health providers because of their proximity [28]. With the biomedical treatment for diabetes not easily accessible and traditional medicine easily accessible, this could be one of the factors that make patients with diabetes use traditional medicine instead of or concurrently with biomedical medicine. This calls for an increase in the number of health facilities that can manage diabetes so as to reduce the distance the patients with diabetes have to travel to seek treatment.

Traditional medicine is easy to use because one can combine it with the biomedical medicine. In addition, unlike with biomedical medicine, one does not need to eat before taking traditional medicine. The convenience of taking traditional medicine is higher than that for biomedical medicine. It is critical that patients with diabetes are educated on the nature of the treatment of diabetes so that they do not see the restrictions in treatment regimes as an unnecessary burden.

Some patients with diabetes were told by traditional healers that traditional herbs can cure diabetes. This could have been a motivator for the patients continuing to seek such care. The relationship of perceived benefit as a motivation for health care seeking is supported by other studies in South Africa and Asia [29-31]. The patients’ beliefs were further strengthened by similar perceptions about herbs as a cure for diabetes within their social networks. Community members encouraged the patients with diabetic symptoms to seek care from traditional healers telling them that the illness will cure. Although beliefs in traditional healers are receding and giving way to biomedical treatment in some parts of Africa [32], many countries still have populations that use traditional medicine (also called alternative medicine) and this happens in low, middle and high income countries [11,33,34]. However the proportion using traditional medicine increases in more remote areas where medical services are thin [35,36]. At the community level, there is need to address the misleading support for traditional herbs as a cure for diabetes. This could be achieved through different fronts. One is sensitizing the community so that the members can identify the symptoms of diabetes as of an illness that is managed in health facilities. Two is educating the patients with diabetes about what to expect when they start treatment so that they may not easily fall prey to people who promise prompt cure with traditional medicine.

### Methodological considerations

The qualitative design was used to explore the reasons why patients with diabetes use traditional medicine. This methodology may not give the magnitude of the practice of using traditional herbs but the qualitative method is preferable for questions that ask the ‘why’. It opens the participants to give reasons for a practice without any restriction to pre-defined options. The limitation of FGDs is that they may not assess the complexity of beliefs nor investigate actual behaviours [37]. Triangulating methods (using KIIs and FGDs) and study participants (patients with diabetes, health workers and herbalists) was very useful to check the consistency and contradictions across and within groups [38]. It was not possible to get the views of the patients with diabetes who had abandoned the biomedical treatment for the traditional medicine individually. This is because traditional medicine is still looked upon in Eastern Uganda as a bad practice and it would be difficult to get patients who would admit taking traditional medicine. However some of the patients with diabetes who were involved in the discussions admitted having used herbs before. The involvement of the herbalists also gave us some insights into what herbalists do to get patients.

## Conclusions

Patients with diabetes use herbs because of community influence, inadequacies in the functionality of the health care system and easy access to herbs. Herbalists are within the community and family members believe that herbs cure diabetes. High costs incurred in accessing biomedical medicine, with health facilities that provide diabetic care being far and sometimes having stock-outs of diabetic drugs, frustrate patients with diabetes. On the other hand, herbalists who live within the community aggressively market herbs in public places. Taking herbs is more convenient as there are no dietary restrictions like in taking biomedical drugs. Payment at herbalists is also convenient as patients can pay in installments. Options of putting diabetic drugs at health facilities that are closer to patients need to be considered. There should be a constant supply of diabetic drugs at facilities that manage patients with diabetes. Community members need to be sensitized on symptoms of diabetes, the need to seek care from health facilities and once diagnosed as being diabetic, adherence to biomedical treatment.

## Competing interests

The authors declare that they have no competing interests.

## Authors’ contributions

ER, LM, SK and FK conceptualized the study, ER, LM, AO and DM were involved in tools development and data collection, ER, LM, SK and DM did the analysis, all the authors were involved in manuscript writing and approved the final manuscript.

## Pre-publication history

The pre-publication history for this paper can be accessed here:

http://www.biomedcentral.com/1472-698X/13/1/prepub
